# RNA-Seq-Based TCR Profiling Reveals Persistently Increased Intratumoral Clonality in Responders to Anti-PD-1 Therapy

**DOI:** 10.3389/fonc.2020.00385

**Published:** 2020-04-28

**Authors:** Ekaterina A. Zhigalova, Anna I. Izosimova, Diana V. Yuzhakova, Lilia N. Volchkova, Irina A. Shagina, Maria A. Turchaninova, Ekaterina O. Serebrovskaya, Elena V. Zagaynova, Dmitriy M. Chudakov, George V. Sharonov

**Affiliations:** ^1^Center of Life Sciences, Skolkovo Institute of Science and Technology, Moscow, Russia; ^2^Laboratory of Genomics of Antitumor Adaptive Immunity, Privolzhsky Research Medical University, Nizhny Novgorod, Russia; ^3^Genomics of Adaptive Immunity Department, Shemyakin and Ovchinnikov Institute of Bioorganic Chemistry, Moscow, Russia; ^4^Molecular Technologies Department, Institute of Translational Medicine, Pirogov Russian National Research Medical University, Moscow, Russia

**Keywords:** tumor-infiltrating lymphocytes, TCR repertoire, RNA-Seq, anti-PD-1, T cell clonality, MiXCR

## Abstract

Substantial effort is being invested in the search for peripheral or intratumoral T cell receptor (TCR) repertoire features that could predict the response to immunotherapy. Here we demonstrate the utility of MiXCR software for TCR and immunoglobulin repertoire extraction from RNA-Seq data obtained from sorted tumor-infiltrating T and B cells. We use this approach to extract TCR repertoires from RNA-Seq data obtained from sorted tumor-infiltrating CD4^+^ and CD8^+^ T cells in an HKP1 (Kras^G12D^p53^−/−^) syngeneic mouse model of lung cancer after anti-PD-1 treatment. For both subsets, we demonstrate decreased TCR diversity in response to therapy. At a later time point, repertoire diversity is restored in progressing disease but remains decreased in responders to therapy in both CD4^+^ and CD8^+^ subsets. These observations complement previous studies and suggest that stably increased intratumoral CD4^+^ and CD8^+^ T cell clonality after anti-PD-1/PD-L1 therapy could serve as a predictor of long-term response.

## Introduction

Active tumor infiltration by CD8^+^ and Th1 T cells has repeatedly been shown to correlate with improved clinical outcomes in a variety of cancers (1–4). At the same time, it remains a matter of debate which proportion of these infiltrating T cells is actually tumor-reactive and could participate in an antitumor response (5), and this proportion may differ between different cancer types and individual patients.

T cell receptor (TCR) repertoire analysis can reveal the clonal content of tumor-infiltrating T cells, the presence of large clonal expansions (6), and the presence of clusters of convergent TCR variants that potentially respond to the same antigen (7–10). However, the prognostic and predictive value of TCR repertoire profiling in cancer immunotherapy remains a matter of investigation.

In a seminal work by Tumeh et al. (6), it was shown that high intratumoral T cell clonality—indicating the presence of large clonal expansions—may be associated with clinical response to anti-PD-1 therapy in patients with advanced melanoma. Furthermore, responders demonstrated a tendency toward increased clonal expansion during therapy. Tamura et al. (11) likewise observed increased intratumoral T cell clonality in response to peptide vaccines and oxaliplatin-based chemotherapy in colorectal cancer patients who exhibited long periods of progression-free survival. A combination of neoadjuvant ipilimumab with high-dose IFNα2b in advanced melanoma showed higher efficiency for patients exhibiting increased T cell clonality in the primary tumor at 6–8 weeks following neoadjuvant therapy (12).

Several studies have also shown that the analysis of peripheral blood TCR repertoire clonality could assist in predicting therapeutic outcomes. In particular, response to anti-PD-1 therapy has been associated with the initial presence of clonal peripheral blood T cell expansions in metastatic melanoma (13), although the opposite was reported for PD-L1 blockade in urothelial cancer (14). In another study of metastatic urothelial cancer patients treated with anti-PD-L1, clinical response was associated with high intratumoral T cell clonality and induced peripheral blood expansion of major tumor-resident T cell clones (15).

Response to anti-CTLA-4 therapy has been linked with initially low peripheral blood TCR clonality in melanoma (13) and pancreatic ductal adenocarcinoma (16), with the latter study also observing increased presence of clonal expansions over the course of therapy (16). These results are in line with the logic of anti-CTLA-4 action via blocking regulatory T cell (T_reg_)-mediated suppression of antigen-presenting cells and interclonal competition between CD4^+^ T cells (17–20). This allows multiple novel expansions to arise, thereby broadening the peripheral TCR repertoire (21). Although anti-CTLA-4 therapy has been associated with essential remodeling and diversification of peripheral TCR repertoires, it has also been reported that improved clinical outcomes may be associated with the persistence of initially high-frequency clones during therapy (22). Using the ALICE algorithm on the data described in Robert et al. (21) and Subudhi et al. (23), we have also recently shown that the number of TCR sequences actively involved in current immune response—as judged by the number of clusters of non-randomly met (non-public) homologous TCR variants—increases after anti-CTLA4 therapy (10), suggesting reactivation of immune response to diverse antigens.

Notably, an increase in intratumoral T cell clonality was also observed in response to targeted therapy with a BRAF inhibitor, and persistence of initially detected dominant T cell clones was associated with therapy response (24). In a B16 mouse melanoma model, expansion of CD8^+^ T cells within the tumor—but not in the periphery—was associated with antitumor effects (25). In FGFR2^K660N^/p53^mut^ lung cancer mouse model, reduced TCR clonality was found in responders receiving anti-PD-1 therapy in combination with an FGFR inhibitor (26). Thus, the current data on the dependence of response to different immunotherapies on the clonal composition of T cell repertoires remain incomplete and somewhat contradictory.

A recent study on the HKP1 (Kras^G12D^p53^−/−^) immunocompetent, syngeneic mouse lung cancer model, which is histologically similar to human adenocarcinoma (27), used RNA-Seq analysis of fluorescence-activated cell sorting (FACS)-sorted tumor-infiltrating CD4^+^ and CD8^+^ T cells in order to reveal the intrinsic features of T cell behavior associated with early immune response to anti-PD-1 therapy (28). This work showed that response to anti-PD-1 treatment was correlated with T cell subset-specific alterations, although the clonality of T cells was not specifically analyzed. However, TCR transcripts are present in bulk RNA-Seq data and enriched in sorted T cell RNA-Seq data, and MiXCR software allows one to extract TCR CDR3 repertoires with near-maximal efficiency and accuracy (29–31).

Here, we show that MiXCR efficiently extracts TCR and immunoglobulin repertoires from RNA-Seq data obtained from sorted tumor-infiltrating T or B cells. We applied this approach to extract TCRα and β CDR3 repertoires from the tumor-infiltrating T cell RNA-Seq data reported by Mittal and colleagues (28), and compared clonality and diversity parameters in responders to anti-PD-1 therapy vs. progressors and untreated control mice. At 1 week after the start of therapy, we find that TCR diversity goes down in both CD4^+^ and CD8^+^ T cells, reflecting clonal expansion. At a later time point, about 2 weeks after the start of the therapy, diversity remains low for responders, but reverts back to high diversity in progressors, reflecting reduced clonal expansion. These data demonstrate that the primary response to anti-PD-1 immunotherapy, as expressed by clonal expansion of T cells, is insufficient to provide sustained response to therapy, and that stability of the intratumoral clonal T cell expansions acquired in the course of the treatment is associated with longer-term response.

## Results and Discussion

RNA-Seq based immune repertoire profiling may work well for sorted T or B cells, where the percentage of CDR3-covering reads is relatively high (30). This approach—preferably using relatively long (e.g., 100+100-nt) paired-end sequencing—makes it possible to combine transcriptomic profiling of tumor-infiltrating lymphocyte populations of interest with relatively deep profiling of immune repertoires.

To illustrate and validate this approach, we performed RNA-Seq analysis on sorted T and B cells from mouse B16F0 melanoma tumors. Tumors were excised and carefully cleaned from the outer fibrous capsule, which contains a large amount of immune cells, in order to focus on lymphocytes that have truly infiltrated the tumor tissue. CD4^+^ T cells or CD19^+^ B cells were sorted from the isolated tumor tissues, and all extracted RNA materials and all obtained cDNA were used to generate cDNA libraries using the Clontech Smart-Seq v4 Ultra Low Input RNA Kit. These were sequenced on an Illumina HiSeq (100+100-nt paired-end reads), and TCRβ or IGH CDR3 repertoires were extracted using the MiXCR software. Table 1 shows typical counts of cells, obtained RNA-Seq reads, CDR3 reads, and CDR3 clonotypes extracted from RNA-Seq data for the sorted T and B cells.

**Table 1:** CDR3 counts from sorted T and B cells from B16F0 melanoma.

**Subset**	**Cells sorted**	**RNA-Seq reads**	**IGH/TCRβ CDR3 containing reads**	**IGH/TCRβ CDR3 clonotypes**
CD19^+^ B cells	6,000–25,000	5–10 million	10,000–50,000	1,000–4,000
CD4^+^ T cells (excluding T_reg_)	8,000–35,000	5–10 million	400–2,000	300–700

**Figure 1:**
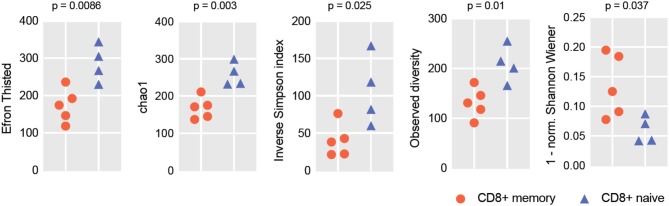
RNA-Seq-based TCR repertoire diversity in naïve and memory CD8^+^ T cells. Diversity metrics calculated for naïve and effector CD8^+^ TCR α + β (combined) CDR3 repertoires are shown. Note that each sample is represented by 1,500 randomly chosen CDR3-covering sequencing reads for normalization. Observed diversity is a total number of unique clonotypes in a sample, so it takes into account all clonotypes. Chao1 depends mostly on the representation of singletons and doubletons—clonotypes represented by one and two reads, respectively. Both Chao1 and Efron-Thisted indices estimate relative total TCR diversity, similar to the estimation of species richness. These numbers should not be, of course, understood as true total diversity, but only as a lower bound calculated based on a given sample size. Inverse Simpson's Diversity Index takes into account both richness and evenness. [1-normalized Shannon Wiener] represents “clonality” metrics used in Tumeh et al. (6) and largely reflects the presence of large clonal expansions.

To verify if the RNA-Seq-based repertoire analysis can be informative in terms of repertoire diversity and clonality, we extracted TCRα and TCRβ CDR3 repertoires from transcriptomes obtained for the human naïve and effector CD8+ T cells reported in Simoni et al. (32). For each sample, we extracted from 399 to 1,255 distinct TCRα and from 795 to 1,579 TCRβ CDR3 clonotypes. Due to the limited repertoire information extracted from some of the samples, we opted to analyze joint TCRα and β repertoires in order to get better averaged statistics for diversity metrics. For normalization (33), we down-sampled our clonesets by extracting an equal number of 1,500 randomly chosen CDR3-covering reads from each set of clones. Diversity and clonality metrics were analyzed using the VDJtools software (34); results are shown in Figure 1. This analysis clearly distinguished naïve and effector CD8+ subsets, as expected.

We next applied the same approach to the CD4^+^ and CD8^+^ sorted T cell RNA-Seq data obtained by Mittal and colleagues from their syngeneic mouse model of lung cancer (28). We extracted joint TCRα and β CDR3 repertoires from these data using the MiXCR RNA-Seq mode. These samples were divided into four groups: (1) controls treated with IgG2a; (2) early response to anti-PD-1 therapy, with therapy on days 7, 10, and 13 and tumors excised at day 14 post-implantation; (3) late response with regression, with therapy on days 7, 10, 13, and 16 and tumors excised at day 17 or 24 post-implantation; and (4) late response with progression, with the same treatment and excision regimen. Note that the early response group was not split into regression and progression due to the insufficient number of usable samples that would have sufficient depth of TCR repertoire analysis in terms of CDR3-covering reads.

Similar to the one described above, we analyzed joint TCRα and β repertoires, down-sampled by extracting an equal number of 500 randomly chosen CDR3-covering reads from each set of clones. Datasets with fewer than 500 reads were discarded.

**Figure 2:**
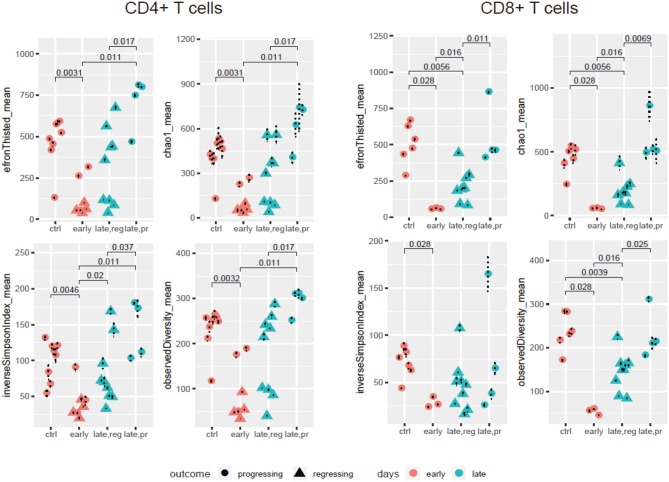
TCR repertoire diversity in the course of anti-PD-1 therapy. Diversity metrics calculated for CD4^+^ and CD8^+^ TCR α + β (combined) CDR3 repertoires extracted from the four groups of samples: control; early response (day 14); late response, regressing (days 17 and 24); and late response, progressing (days 17 and 24). Each sample is represented by 500 randomly chosen CDR3-covering sequencing reads, for normalization.

Our comparison of the resulting diversity and clonality metrics yielded several findings (Figure 2, Supplementary Figure 1). First, the diversity of TCR repertoires in anti-PD-1-treated animals decreased at early time points compared to controls treated by IgG2a, both in the CD4^+^ and CD8^+^ subsets. Additionally, although the limited number of samples did not allow us to estimate statistically meaningful differences between responders and progressors at the early time point (day 14), there was a trend toward more reduced CD4^+^ diversity among the responders. At the later time points (day 17 or 24), repertoire diversity was restored in both progressors and responders, and in both CD4^+^ and CD8^+^ subsets. However, the latter effect was much stronger for progressors, leading to statistically significant differences in repertoire diversity metrics between responders and progressors at the late time point for both T cell subsets.

These data complement the results obtained by Mittal and colleagues as well as the current knowledge on the association between TCR clonality and response to anti-PD-1 therapy. Sustained response is associated with stably increased clonality and decreased diversity in both CD4^+^ and CD8^+^ subsets over the course of therapy, while restoration of initial diversity seems to be associated with disease progression.

One possible interpretation is that initial proliferation of previously suppressed PD-1^+^ T cell clones specific to locally present immunogenic antigens—including both tumor and non-tumor (e.g., viral) antigens (32, 35)—in response to anti-PD1 therapy leads to temporarily increased clonality and decreased diversity. Notably, it has been shown that increased intratumoral TCR clonality in adenocarcinoma is uniquely correlated with CD8^+^PD-1^+^ T cell subsets, but not with bulk CD8^+^ T cells (36). The same observation was noted in targeted analysis of CD8^+^PD1^+^ T cell subsets in NSCLC after anti-PD-1 treatment. Only intratumoral CD8^+^ T lymphocyte populations with high PD-1 expression levels were characterized as having significantly increased clonality (37). Thus, the pool of oligoclonal PD-1^+^ T cells could serve as a source for rapidly growing clonality in response to anti-PD-1 therapy.

On the other hand, some studies show that PD-1 blockade may unleash a novel tumor-specific TCR repertoire that was not previously observed in the tumor (38). Experiments tracking tumor-infiltrating TCR clones from patients with basal cell carcinoma and squamous cell carcinoma have demonstrated that clonal expansions of memory CD8^+^ T cells with an exhausted phenotype referred to chronical activation before and after anti-PD-1 therapy are distinct from each other. The authors called this phenomenon “clonal replacement” and suggested that exhaustion of tumor-infiltrating T cells limits their renewal following checkpoint blockade. They further proposed that the T cell response relies on the expansion of novel tumor-specific T cell clones originating from non-tumor sites such as lymphoid organs or rare, unexpanded clones present within the tumor (38).

The association of a prolonged response to treatment with the stability of clonal expansions acquired during initial response, as we have observed in this work, makes intuitive sense and may be interpreted as evidence that a sustained immune response results in the stable presence and proliferation of tumor-specific CD4^+^ and CD8^+^ T cells.

Our results support the concept that monitoring intratumoral T cell clonality—for example, by measuring T cell clonality in excised tumor samples after neoadjuvant anti-PD-1/PD-L1 therapy (6, 11, 12, 39)—is a rational strategy for predicting long-term response.

Technically, we advocate for performing RNA-Seq on sorted T and B cell subsets as a means to simultaneously evaluate both functional behavior and repertoire features in response to immunotherapy. MiXCR currently provides the most efficient computational solution for such analyses, either as an offline tool (https://mixcr.milaboratory.com) or as an Illumina BaseSpace cloud application (https://www.illumina.com/products/by-type/informatics-products/basespace-sequence-hub/apps/milaboratory-mixcr-immune-repertoire-analyzer.html).

## Methods

### Mouse Melanoma B16F0 Tumor Model

Experiments were carried out on C57BL/6-Foxp3^eGFP^ mice kindly provided by Prof. A. Rudensky (Memorial Sloan-Kettering Cancer Center) (40). Tumors were generated by subcutaneous (s.c.) injection of 10^5^ B16F0 cancer cells in 300 μl PBS into the left flank of 5–7-month-old female mice. These tumor cells were initially grown in a DMEM medium supplemented with 10% fetal bovine serum (FBS), 0.06% L-glutamine, 50 units/ml penicillin, and 50 μg/ml streptomycin. Cells were cultured in an incubator at 37°C and 5% CO_2_ and passaged two or three times per week. Before injection, cells were detached by trypsin, then counted and resuspended in PBS to a final concentration of 10^6^ cells in 3 ml. Mice with tumor diameters ranging from 0.5 to 1.2 cm were sacrificed with isoflurane (Esteve, Italy), after which tumors were excised and prepared for further analysis. All animal experiments were carried out in accordance with the National Institutes of Health Guide for the Care and Use of Laboratory Animals (NIH Publications No. 8023, revised 1978). The experimental protocol was approved by the Ethical Committee of the Privolzhsky Research Medical University Academy, Russia (EC #6, granted April 17, 2019).

### Mouse Melanoma Resection and Lymphocyte Isolation

Tumors were excised and cleaned from the outer tumor capsule. For lymphocyte isolation, excised tumor nodules or tumor parts were homogenized with a gentleMACS dissociator (Miltenyi Biotec, Germany) and incubated in 1–2 ml dissociation solution [RPMI medium supplemented with 417 μg/ml Liberase TL (Roche, Germany) and 10 μg/ml DNase I (Roche, Germany) for 30 min at 37°C in a shaker]. After dissociation, cell suspensions were passed through a 70-μm cell strainer and washed twice with 5 ml of an incubation buffer (PBS, pH 7.2 containing 0.5% bovine serum albumin and 2 mM EDTA).

Cell pellets were resuspended in 100 μl of an incubation buffer with the following antibodies (2 μl each): CD45-PerCP/Cy5.5 (Clone 30-F11, BioLegend), CD3-APC (Clone 145-2C11, BioLegend), CD4-V450 (Clone RM4-5, BD Biosciences), and CD19-PE/Cy7 (Clone 6D5, BioLegend); 400 μl of incubation buffer was added after 45–120 min staining at 4°C. CD3^+^CD4^+^ (excluding eGFP-positive T_reg_ cells) and CD19^+^ subsets were sorted with a FACSAria III cell sorter (BD Biosciences) using the 85-μm nozzle directly into 200 μl of an RLT cell lysis buffer (Qiagen). After sorting, the samples were vortexed and then left at room temperature for 10 min to ensure cell lysis, and finally stored at −20°C.

### Mouse Model of Non-small-cell Lung Cancer

Mittal and colleagues utilized an immunocompetent, syngeneic preclinical model of early-stage non-small-cell lung cancer (NSCLC), which has been shown to exhibit histological similarities to human adenocarcinoma (28). Mice were injected with 250 μg of a rat monoclonal blocking anti–PD-1 antibody or IgG2a intraperitoneally on days 7, 10, 13, and 16 after tumor implantation. Mice were sacrificed on day 14, day 17, or day 24 depending on the group. RNA was extracted from sorted tumor-infiltrating CD4^+^ and CD8^+^ T cells. RNA-Seq libraries were prepared with an Illumina TruSeq RNA Sample Preparation kit and sequenced on an Illumina HiSeq4000 with single-end 50-bp reads, 8 samples per lane. Samples were grouped based on the day of tumor excision (day 14—early tumors, days 17 and 24—late tumors) and tumor growth (progressing, regressing, or partially regressing). For reference, see Figure 7A in Markowitz et al. (28). There were 31 samples of intratumoral CD8^+^ and CD4^+^ T cells in total (Supplementary Table 1). Joint TCRα and β CDR3 repertoires were extracted from raw fastq files using MiXCR v2.1.11 (https://github.com/milaboratory/mixcr/releases/tag/v2.1.11).

## Data Availability Statement

Publicly available datasets were analyzed in this study. This data can be found here: The RNA sequencing data are available in the Gene Expression Omnibus database (http://www.ncbi.nlm.nih.gov/gds) under the accession number GSE114300.

## Ethics Statement

All animal experiments were carried out in accordance with the National Institutes of Health Guide for the Care and Use of Laboratory Animals (NIH Publications No. 8023, revised 1978). The experimental protocol was approved by the Ethical Committee of the Privolzhsky Research Medical University Academy, Russia (EC #6, granted April 17, 2019).

## Author Contributions

EZh, MT, and DC worked on data analysis. AI, DY, LV, IS, and GS worked with mouse tumors and library preparation. ES, MT, EZa, DC, and GS wrote the manuscript. EZa and DC supervised the study.

## Conflict of Interest

DC is a co-founder of MiLaboratory LLC, which develops MiXCR software and has exclusive rights for its commercial distribution. The remaining authors declare that the research was conducted in the absence of any commercial or financial relationships that could be construed as a potential conflict of interest.
