# Supplementary material for: RNA-Seq-Based TCR Profiling Reveals Persistently Increased Intratumoral Clonality in Responders to Anti-PD-1 Therapy
Source: Front Oncol. 2020 Apr 28;10:385. doi: 10.3389/fonc.2020.00385 (PMC7199218; doi:10.3389/fonc.2020.00385)
Supplement: Supplementary file 1 [file Image_1.pdf]

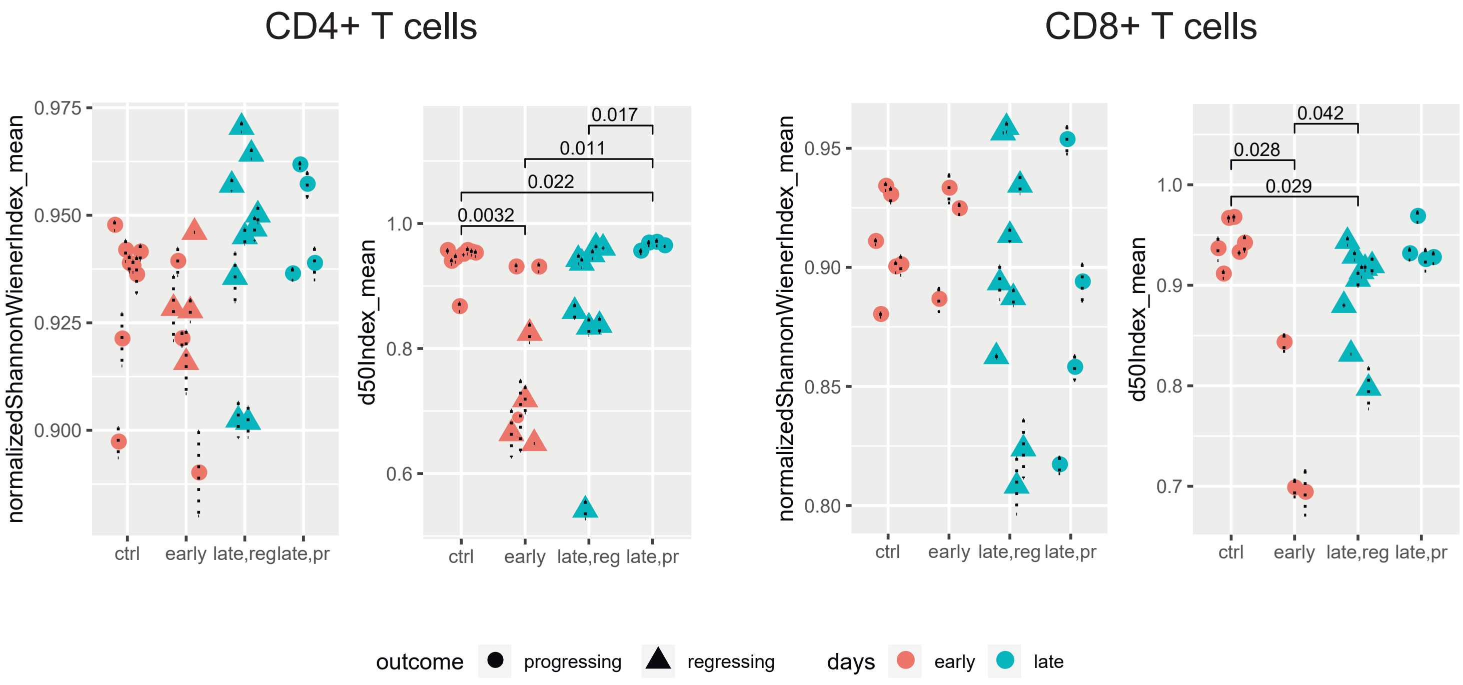

Supplementary Figure 1. Normalized Shannon Wiener and d50 TCR repertoire diversity index in the course of anti-PD1 therapy. Note that normalized Shannon-Wiener index did not distinguish selected groups, indicating that estimation of repertoire clonality based on unevenness focused on the largest clonal expansions may in some cases lose useful information on repertoire diversity provided by other diversity metrics.
