# Supplementary material for: RNA-Seq-Based TCR Profiling Reveals Persistently Increased Intratumoral Clonality in Responders to Anti-PD-1 Therapy
Source: Front Oncol. 2020 Apr 28;10:385. doi: 10.3389/fonc.2020.00385 (PMC7199218; doi:10.3389/fonc.2020.00385)
Supplement: Supplementary file 2 [file Table_1.pdf]

Supplementary Table 1. Analyzed RNA-Seq samples from tumour-infiltrating CD4$^+$ and CD8$^+$ T cells reported in Ref: (Markowitz et al., 2018).

sample	sample ID, CD8 T cells	sample ID, CD4 T cells	treatment	tumour growth	day of tumour excision	time point
24_7	SRR7145378	SRR7145347	anti-PD-1	progressing	24	late
24_6	SRR7145377	SRR7145346	anti-PD-1	progressing	24	late
17_5	SRR7145376	SRR7145345	anti-PD-1	progressing	17	late
17_1	SRR7145375	SRR7145344	anti-PD-1	progressing	17	late
24_8	SRR7145374	SRR7145343	anti-PD-1	regressing	24	late
24_5	SRR7145373	SRR7145342	anti-PD-1	regressing	24	late
24_3	SRR7145372	SRR7145341	anti-PD-1	regressing	24	late
24_4	SRR7145371	SRR7145340	anti-PD-1	regressing	24	late
24_2	SRR7145370	SRR7145339	anti-PD-1	regressing	24	late
24_1	SRR7145369	SRR7145338	anti-PD-1	regressing	24	late
17_8	SRR7145368	SRR7145337	anti-PD-1	regressing	17	late
17_7	SRR7145367	SRR7145336	anti-PD-1	regressing	17	late
17_6	SRR7145366	SRR7145335	anti-PD-1	regressing	17	late
17_4	SRR7145365	SRR7145334	anti-PD-1	regressing	17	late
17_3	SRR7145364	SRR7145333	anti-PD-1	regressing	17	late
14P_3	SRR7145363	SRR7145332	anti-PD-1	progressing	14	early
14P_2	SRR7145362	SRR7145331	anti-PD-1	progressing	14	early
14P_8	SRR7145361	SRR7145330	anti-PD-1	progressing	14	early
14P_7	SRR7145360	SRR7145329	anti-PD-1	progressing	14	early
14P_6	SRR7145359	SRR7145328	anti-PD-1	regressing	14	early
14P_5	SRR7145358	SRR7145327	anti-PD-1	regressing	14	early
14P_4	SRR7145357	SRR7145326	anti-PD-1	regressing	14	early
14P_1	SRR7145356	SRR7145325	anti-PD-1	regressing	14	early
14I_8	SRR7145355	SRR7145324	IgG2a	progressing	14	control
14I_7	SRR7145354	SRR7145323	IgG2a	progressing	14	control
14I_6	SRR7145353	SRR7145322	IgG2a	progressing	14	control
14I_5	SRR7145352	SRR7145321	IgG2a	progressing	14	control
14I_4	SRR7145351	SRR7145320	IgG2a	progressing	14	control
14I_3	SRR7145350	SRR7145319	IgG2a	progressing	14	control
14I_2	SRR7145349	SRR7145318	IgG2a	progressing	14	control
14I_1	SRR7145348	SRR7145317	IgG2a	progressing	14	control

Reference:

Markowitz, G.J., Havel, L.S., Crowley, M.J., Ban, Y., Lee, S.B., Thalappillil, J.S., Narula, N., Bhinder, B., Elemento, O., Wong, S.T., Gao, D., Altorki, N.K., and Mittal, V. (2018). Immune reprogramming via PD-1 inhibition enhances early-stage lung cancer survival. *JCI Insight* 3.
